# Laparoscopy-assisted transgastric endoscopic retrograde cholangiopancreatography for choledocholithiasis after Roux-en-Y gastric bypass: A case report

**DOI:** 10.1016/j.amsu.2019.06.008

**Published:** 2019-06-14

**Authors:** Mauricio Gonzalez-Urquijo, Adrian A. Baca-Arzaga, Eduardo Flores-Villalba, Mario Rodarte-Shade

**Affiliations:** a Tecnologico de Monterrey, Escuela de Medicina y Ciencias de la Salud, Dr. Ignacio Morones Prieto O 3000, Monterrey, 64710, Mexico; b Tecnologico de Monterrey. Escuela Nacional de Ingeniería. Departamento de Ciencias Clinicas. Hospital Zambrano Hellion, Batallon de San Patricio 112, Col. Real de San Agustin, Monterrey, 66278, Mexico

**Keywords:** LA-ERCP, RYGB, Biliary disease, Gallstones, Obesity

## Abstract

**Background:**

Exclusion of the stomach after Roux-en-Y gastric bypass (RYGB) makes access to the biliary tree very challenging for the surgeon or the endoscopist. Different techniques have been described to overcome this downside, including laparoscopy-assisted transgastric endoscopic retrograde cholangiopancreatography (ERCP), which is an outstanding method to access the remnant stomach in order to reach the duodenal papilla. The use of this technique is associated with a high success rate.

**Presentation of case:**

Here we present the case of a 57-year-old patient with altered RYGB anatomy. The patient underwent laparoscopic cholecystectomy. Intraoperative cholangiography revealed the presence of a stone in the common bile duct. A laparoscopy-assisted transgastric ERCP was performed successfully. During the procedure, the duodenoscope was introduced through a gastrostomy, obviating the need for an intragastric trocar. The patient evolved favorably and was discharged on second postoperative day without any complications.

**Discussion:**

Transgastric laparoscopy-assisted ERCP represents an effective approach for the management of biliary complications after RYGB, even if there is a long interval between the two interventions, as occurred in the present case. Other methods described for accessing the biliary tree in patients with altered RYGB anatomy are double-balloon ERCP and endoscopic ultrasound-directed transgastric ERCP. We elected to perform the laparoscopy-assisted approach because choledocholithiasis was diagnosed transoperatively, thus, avoiding the need for secondary procedures or interventions.

**Conclusion:**

Transgastric laparoscopy-assisted ERCP is a feasible procedure with low complication rates and is used in treating patients with altered RYGB anatomy who present with biliary tract disorders. The use of transgastric laparoscopy-assisted ERCP allows endoscopic treatment and cholecystectomy to be performed in a single setting.

## Highlights

• Case report of a patient with altered RYGB anatomy, which presented choledocholithiasis.
• There are several ways to treat this disorder on patients with this condition.
• Laparoscopic Assisted Trans-gastric ERCP is a feasible and secure option.

## 1 Introduction

Among all bariatric surgical procedures, Roux-en-Y gastric bypass (RYGB) is considered the “gold standard” for treating the well-known obesity pandemic. The use of this procedure has increased in the last two decades, causing an exponential increase in the prevalence of RYGB anatomy and the frequency with which operating surgeons encounter pathologies that require treatment through the native stomach, such as pancreatobiliary diseases. It is estimated that 36% of patients who undergo RYGB develop gallstones. Among these patients, 5.3% require pancreaticobiliary interventions. Therefore, physicians must identify methods that can be used to access the biliary tree, such as laparoscopy-assisted transgastric endoscopic retrograde cholangiopancreatography (LA-ERCP) [1].

Here we present the case of a 57-year-old male with RYGB-altered anatomy who presented with choledocholithiasis and was subsequently treated successfully with LA-ERCP at an academic teaching institution. Furthermore, we present a review of the relevant literature. The work has been reported in line with the SCARE criteria [2].

## 2 Presentation of Case

A 57-year-old male patient who had no remarkable family history and had a history of diabetes mellitus and a 10-year history of laparoscopic RYGB for morbid obesity (BMI 55 kg/m^2^) who was not taking any drugs and was a non-smoker was admitted to the hospital with a 1-month history of intermittent colicky mild abdominal pain that had been progressing over several days with nausea and vomiting. The patient did not exhibit any signs of jaundice, choluria, or acholia. On examination, he was hemodynamically stable and afebrile. Physical examination revealed abdominal distension with decreased bowel movements to auscultation, accompanied by diffuse tenderness to superficial and deep palpation, with a (+) Murphy sign and a tympanic colonic margin to percussion.

The routine laboratory test results revealed normal hemoglobin, 14.7 g/dL; white blood cell count, 9.7 × 10^10^; total bilirubin, 3.4 mg/dL; direct bilirubin, 2.23 mg/dL; aspartate aminotransferase, 408 U/L; alanine aminotransferase, 315 U/L; and gamma-glutamyl transferase 226 U/L. The results of all other laboratory tests were within normal limits.

**Fig. 1:**
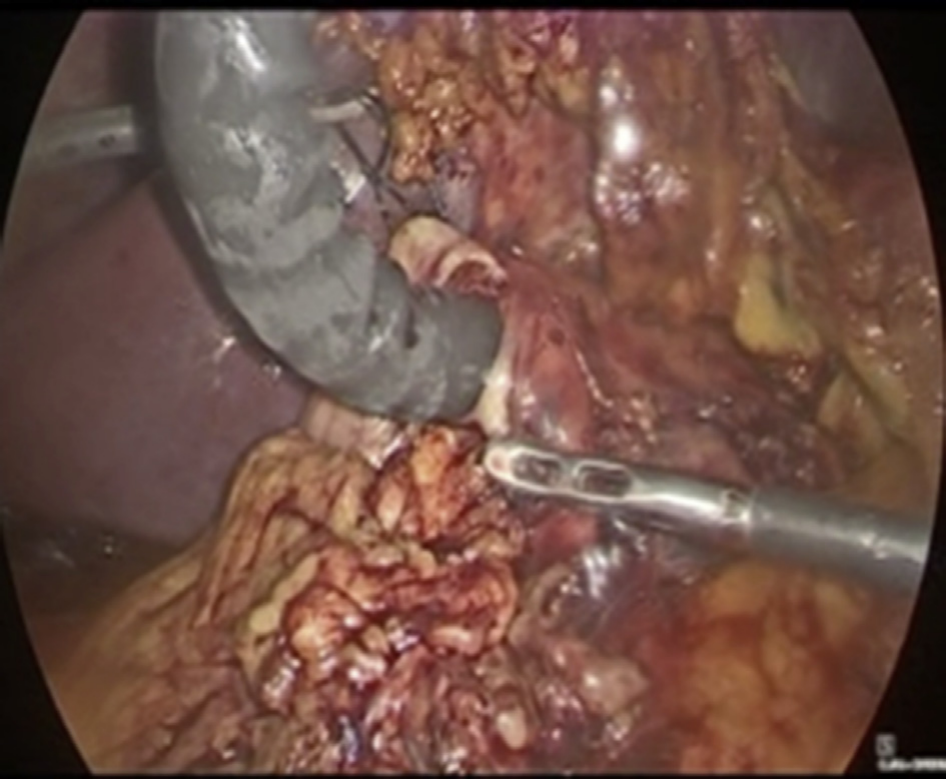
After a single stitch is placed 2 cm above the gastrostomy to pull the stomach up to the abdominal wall, the endoscope is inserted.

**Fig. 2:**
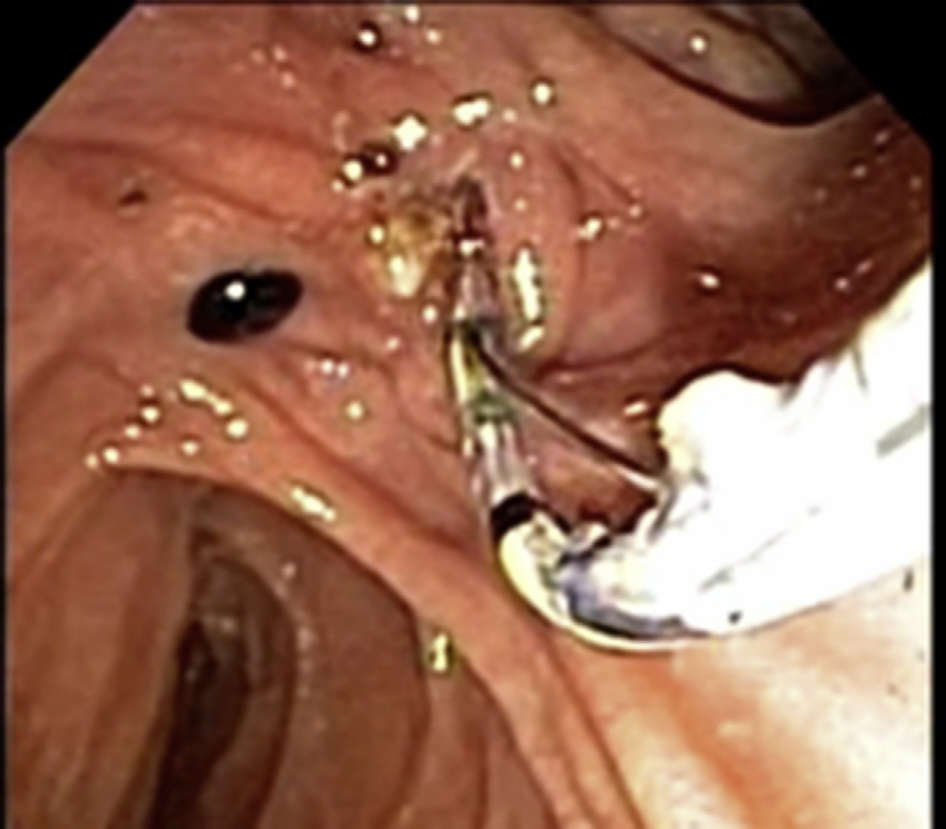
A retrieval balloon catheter is inserted through the ampulla to access the common bile duct.

**Fig. 3:**
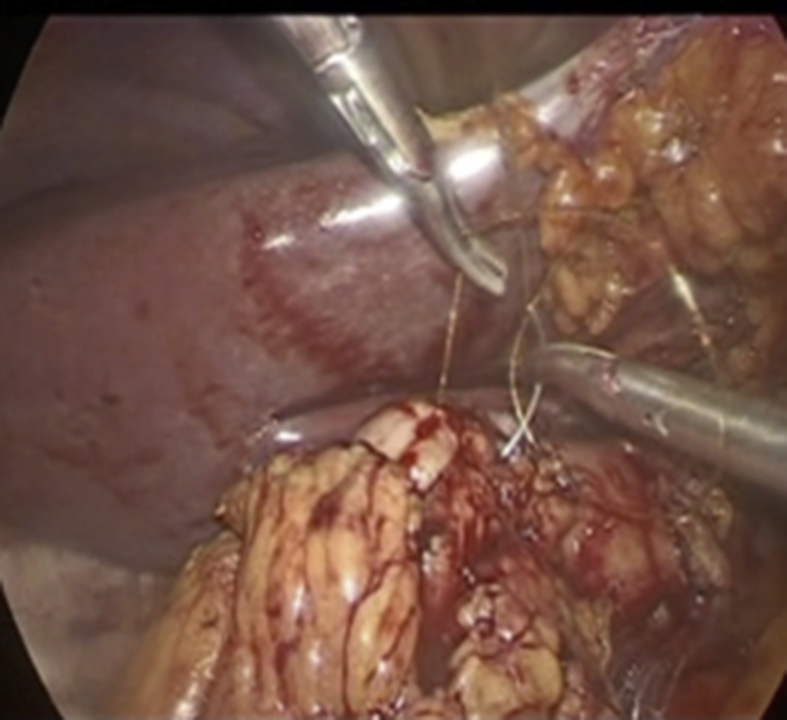
The gastrostomy is closed with a double layer of running resorbable sutures.

Abdominal ultrasonography revealed no dilation of the intrahepatic bile duct and a common bile duct (8 mm) with no apparent gallstones. The gallbladder was distended, with no wall thickening, and contained numerous gallstones. The patient was transferred to the operating room. The senior attending surgeon (who is both a general surgeon and a gastrointestinal endoscopist) placed four trocars following the conventional procedure for performing a cholecystectomy. Calot's triangle was dissected without abnormalities, and a transoperative cholangiogram was performed to visualize a 5-mm stone in the distal common bile duct. The cholecystectomy was completed successfully. With the gallbladder *ex situ*, LA-ERCP was initiated. A 15-mm port was inserted through the abdominal wall to replace the 10-mm trocar in the epigastrium. A single purse-string suture was placed at the greater curvature of the excluded stomach, at the antrum. A 3-cm gastrostomy was created with monopolar electrocautery, and a side-viewing endoscope (Olympus TJF 160 VR or TJF 145) was inserted through the gastric incision, while lifting the suture up against the abdominal wall, to create traction on the stomach, without suturing it to the peritoneum (Fig. 1). The endoscope was advanced through the pylorus until the papilla. Cannulation of the common bile duct was achieved by performing a cholangiogram documenting the initial diagnosis; sphincterotomy was accomplished, and a retrieval balloon catheter was used to remove the stones and biliary sludge from the common bile duct (Fig. 2). The results of cholangiography showed no evidence of any filling defect. The gastrostomy was closed with a double layer of running resorbable sutures, without any complications (Fig. 3; Supplemental Video 1).

Supplementary video related to this article can be found at https://doi.org/10.1016/j.amsu.2019.06.008

The following is the supplementary data related to this article:

Video 1 Video

The patient had a satisfactory postoperative outcome, with only moderate pain on the first postoperative day (POD). On the third POD, the patient was discharged, with good oral intake, normal evacuations, and normal liver enzyme levels. At the 12-month follow-up examination, the patient was doing well, with no biliary symptoms; he was satisfied with the treatment he received.

## 3 Discussion

It has been demonstrated that extreme weight loss resulting from gastric bypass is associated with an increased risk of developing biliary disorders. In a recent monocentric prospective study by Coupaye et al. [3], a 30-kg weight loss >6 months after RYGB was found to be significantly associated with the development of gallstones [3]. These results are similar to those reported by Weinsier et al. [4], who found that a rate of weight loss >1.5 kg per week was associated with the development of cholecystolithiasis. These findings have led to the advocacy by some surgeons for routine prophylactic cholecystectomy when performing RYGB, despite the evidence that this practice shows no benefits over elective approaches [5,6].

The treatment of patients who develop biliary complications, such as choledocholithiasis or cholangitis, after RYGB requires specific technical approaches to successfully reach the biliary tract. Twenty years ago, Baron and Vickers [7] reported the groundbreaking development of a technique in which they introduced the endoscope through a gastrostomy in the greater curvature of the stomach or the remnant pouch, thus, bypassing the distorted gastrointestinal anatomy that results due to a gastric bypass procedure. This led to the development of a new technique that revolutionized the management of biliary stones in patients with RYGB, known as transgastric laparoscopy-assisted endoscopic retrograde cholangiopancreatography (LA-ERCP).

Oral endoscopic management for treating biliary track complications in patients with Roux-en-Y gastrojejunostomy can be performed with double-balloon ERCP, which was designed specifically for deep intubation of the small bowel. The long type of double-balloon endoscopy comprises a 200-cm endoscope with a 145-cm soft overtube, with latex balloons attached to the end of the endoscope and to the end of the overtube. This device allows access to a bilio-enteric anastomosis or papilla through long limbs in patients with altered anatomy [8]. Although less invasive than other techniques, this approach has a success rate of only 60%–70% in the hands of experienced endoscopists [9]. Furthermore, physicians who elect to use this technique encounter technical difficulties such as a decreased accessory performance due to the small diameter of the working channel. The physician may find it difficult to obtain a caudal view of the duodenum or to reach the ampulla of Vater in case of a long Roux limb [10]. A technique to eliminate some of these challenges is endoscopic ultrasound-directed transgastric ERCP (EDGE), which involves the creation of a fistulous tract by placing a lumen-apposing metal stent (LAMS) between either the jejunum or gastric pouch and the excluded stomach under endoscopic ultrasound guidance. The physician subsequently performs conventional ERCP through LAMS. However, clinicians have been unenthusiastic to adopt EDGE due to the concern for the development of a persistent fistula after stent removal, which results in weight regain and exacerbates glycemic control [11]. Another alternative for the management of common bile duct stones found during laparoscopic cholecystectomy could be laparoscopic common bile duct exploration (LCBDE). In a prospective randomized controlled trial performed by Barreras et al. [12], intraoperative ERCP in patients with unaltered anatomy was associated with a higher rate of choledocholithiasis clearance, shorter length of hospital stay and operative time, and lower morbidity than LCBDE, although significant differences were found only for the length of hospital stay (1.2 vs 3.1 days, p < 0.012) and mean operative time (94 vs 117 min, p < 0.001). Additionally, to the best of our knowledge, LA-ERCP has not been compared with LCBDE in patients with RYGB in previous studies. We believe that if an experienced endoscopist is performing the procedure, LA-ERCP may be better than LCBDE for the abovementioned reasons; however, the latter can be performed without any significant differences in postoperative outcomes from the former if the surgery is being performed by a general surgeon with advanced laparoscopic skills.

Following RYGB, not only there is alteration of the anatomy but also there are physiological changes that account for the high incidence of gallstones in these patients. The three main factors involved in gallstone formation are (1) hypersaturation of cholesterol in the bile, (2) gallbladder hypomobility [13], and (3) increased secretion of mucin, which acts as a nucleation factor, in bile [14]. Notably, our patient had a long interval between RYGB and biliary complications (10 years) compared with the mean interval of 12.6 ± 4.3 months described by other authors; this discrepancy may be associated with variable rates of weight loss [15].

In the case presented above, we opted against performing prophylactic cholecystectomy concomitantly with RYGB on the basis of the evidence first published 10 years ago; instead, we elected for prophylaxis with ursodeoxycholic acid (UDA) to prevent biliary disease [16]. Although this approach remains controversial, a recent meta-analysis has shown an increased risk of postoperative complications and an average increase of 32.8 min in the operative time when cholecystectomy is performed simultaneously with RYGB [15]. This extended time in the operating room, in combination with the additional measures required to manage postoperative complications, substantially increases the cost of weight-loss surgery, which is typically shouldered by the patient. UDA prophylaxis has been shown to reduce the incidence of biliary complications, supporting our initial choice [17].

Transgastric LA-ERCP represents an effective approach for the management of biliary complications after RYGB, even if there is a long interval between the two interventions. Banerjee et al. [15] have reported an overall success rate of 98.5% using ERCP through a transgastric approach. This rate is comparable with that achieved with ERCP in patients with standard anatomy. Access to the ampulla was achieved in 98.9% of cases and biliary cannulation in 98.5%; nevertheless, 14% of cases involved an adverse event, and the most common complications were infection of the gastrostomy and ERCP-induced pancreatitis [15].

In our patient, we used a technique similar to that described by Falcao et al. [18], which requires a single purse-string suture 2 cm above the gastric incision to achieve traction, in order to facilitate endoscope insertion. This approach may be held in contrast with that described by Fachiano et al. [19] The authors report lifting the stomach and suturing it to the abdominal wall while introducing the endoscope through a trocar within the stomach. The use of this maneuver is thought to prevent the leakage of gastric contents into the abdominal cavity; however, the scientific literature currently lacks sufficient data to support its use. Using our approach, the endoscope is passed directly through the gastrostomy without the need for an intragastric trocar, avoiding the need to extend the duration of surgery, without any postoperative complications. Table 1 presents diverse approaches, published from 2010 to the current day.

**Table 1:** Transgastric laparoscopy-assisted ERCP in Roux-en-Y gastric bypass patients after 2010.

Author	No. of cases	Age	Female Sex	Procedure time (min)	Port inserted into gastric pouch	Cannulation rate	Conversion to laparotomy	Length of stay (days)	Complication rate overall	Interval RYGBP to ERCP (months)	Notes
Borel, 2019	1	79	100%	–	Yes	100%	0%	16	100%	24	Acute kidney injury
Habenicht Yancey, 2018	16	55.8	–	–	Yes	94%	6%	3.7	6%	82.8	Pancreatitis
Espinel, 2017	2	53	100%	–	No	100%	0%	4	50%	48	Mild pancreatitis
Frederiksen, 2017	29	46	86%	–	Yes	100%	6%	2	35%	–	Hematoma, wound dehiscence, pancreatitis, abscess, perforation of gastric remnant
Manassa, 2016	2	48	100%	–	Yes	100%	0%	4	0%	24	
Paranandi, 2016	7	44	100%	94	Yes	100%	0%	2	28.50%	27.3	Mild pancreatitis, port site infection
Bowman, 2016	15	48.5	73%	–	Yes	100%	6%	3.4	6%		Incisional hernia
Mejia, 2016	4	51	50%	105	Yes	100%	0	2.7	0%	50.4	
Farukhi, 2016	7	–	–	72	Yes	100%	0	1.1	0%	–	
Snawaert, 2015	23	54	78%	40.6	Yes	100%	8.70%	2.8	0	–	
Sun, 2015	22	–	–	156	–	95%	5%	2.6	4.50%		Wound infection
Brockmeyer, 2015	8	*	*	*	Yes	100%	*	*	0%	*	
Melero, 2015	1	34	0%	165	Yes	100%	0%	1	0%	12	
Grimes, 2014	38	47.8	95%	265	Yes	95%	2.60%	4.2	13%	–	
Lin, 2014	8	*	–	187	Yes	100%	0	2.25	0%	–	
Vilallonga, 2013	1	48	100%	70	Yes	100%	0%	4	0%	36	
Schriner, 2012	24	52	79%	172	No	100%	13%	1.67	8.30%	–	Mild pancreatitis, enterocutaneous fistula
Falcao, 2012	23	35.3	82%	92	No	100%	0	2	4%	16.3	Mild pancreatitis
Saleem, 2012	15	50.8	80%	45	Yes	100%	14%	3.5	0%	–	
Bertin, 2011	21	–	–	236	Yes	100%	4%	2.4	14%		Hematoma, biliary leak, retroperitoneal perforation
Faulk, 2011	14	–	–	–	Yes	100%	14%	3.8	14%	–	Enterotomy proximal limb, PO pulmonary embolism
Badaoui, 2010	1	47	100%	90	No	100%	0	3	0%	36	

Not mentioned:

Not specified: *.

PO, postoperative; ERCP, endoscopic retrograde cholangiopancreatography; RYGB, roux-en-Y Gastric bypass.

Values expressed in means and percentages.

With the increasing use of laparoscopic and robotic surgical approaches and the development of new technologies, bariatric surgeons are seeking to incorporate minimally invasive techniques into their repertoire for treating RYGB-related complications. The technique described in the present case report represents additional data in support of using LA-ERCP for the management of biliary complications after RYGBP. Laparoscopic evaluation of the abdominal contents can be performed simultaneously to rule out other causes of abdominal pain, such as internal herniation, which can mimic biliary symptoms.

## 4 Conclusion

With the current obesity epidemic and the popularity of RYGB, the prevalence of altered gastrointestinal anatomy is not uncommon. Laparoscopy-assisted transgastric ERCP is a feasible and secure procedure with a low complication rate for treating patients with altered RYGB anatomy who present with biliary tract disorders. This approach allows for endoscopic treatment to access the biliary tree and cholecystectomy to be performed in a single setting, reducing the total procedure cost, preventing the need for an additional stage of treatment, and decreasing the incidence of postoperative complications.

## Ethical approval

NA.

## Sources of funding

None funding

## Author contribution

Mauricio Gonzalez- Urquijo: He is a third year general surgery resident. He was the leader of the work, he design the case report. He recollected data, and wrote the manuscript.

Adrian Baca: he helped with the manuscript and the final version of it.

Eduardo Flores Villalba: He is a general surgery attending, he helped with the writing of the manuscript.

Mario Rodarte-Shade: He is a general surgery attending, he supervised and opérate on the patient, and he helped with the case report, and the final versión of it.

## Conflicts of interest

None.

## Trial registry number

NCT03925766.

## Guarantor

Mario Rodarte-Shade.

## Consent

Consent has been obtained.

## Provenance and peer review

Not commissioned, externally peer reviewed.
